# Clinimetric evaluation and clinical outcomes of the Dutch version of the Chronic Ear Survey

**DOI:** 10.1186/s12955-019-1173-2

**Published:** 2019-06-18

**Authors:** S. Geerse, R. J. de Haan, M. J. F. de Wolf, F. A. Ebbens, E. van Spronsen

**Affiliations:** 1 Department of Otorhinolaryngology, Amsterdam UMC, University of Amsterdam, Meibergdreef 9, Amsterdam, Netherlands; 2 Clinical Research Unit, Amsterdam UMC, University of Amsterdam, Meibergdreef 9, Amsterdam, Netherlands

**Keywords:** Surveys and questionnaires, Translations, Outcome assessment, Quality of life, Otitis media, Suppurative

## Abstract

**Background:**

To validate and evaluate the reliability of the Dutch version of the Chronic Ear Survey (CES) in patients suffering from Chronic Suppurative Otitis Media (CSOM) and to evaluate clinical outcomes of surgery using this questionnaire.

**Methods:**

We developed the Dutch version of the CES (D-CES) using forward-backward translation of the original CES into the Dutch language. Next, patients with CSOM and controls completed the D-CES pre- and postoperatively. Internal consistency, test-retest reliability, known-group validity and convergent validity were evaluated. In addition to the D-CES, the Short Form 36 (SF-36) was administered to all participants to correlate D-CES data to quality of life.

**Results:**

A total of 29 patients with CSOM scheduled for ear surgery were included. Our control group consisted of 26 patients scheduled for eye surgery, all without signs and symptoms of CSOM. Cronbachs’ α of the complete questionnaire was 0.69. The Intraclass Correlation Coefficients (ICCs), reflecting test-retest reliability, ranged between 0.69 and 0.82. Scores differed significantly between CSOM patients and controls with substantial lower (more impaired) D-CES scores in the CSOM group. Duration of complaints preoperatively and the presence of a dry ear and/or improvement of hearing postoperatively all had a significant impact on D-CES improvement scores. Small to moderate correlations were found between D-CES subscales and matching subscales of the SF-36.

**Conclusion:**

The D-CES is an appropriate disease specific questionnaire to assess a patient’s perceived functional health in CSOM.

## Background

Patient reported outcome measures (PROM’s) and impact of medical and/or surgical treatment on quality of life are of increasing importance in current medical practice, including the field of otology [1–4]. In addition to objective measures such as pure tone audiometry and Computer Tomography (CT), PROM’s provide supplemental information regarding the impact of symptoms on patient burden and patient’s perception of treatment efficacy [5]. Therefore the need for clinimetrically solid patient reported outcome measures is paramount. Koenraads et al. showed that especially the field of otology lacks validated questionnaires [6]. For the Dutch population, only a few questionnaires addressing Chronic Suppurative Otitis Media (CSOM) are available [1, 4]. As CSOM effects approximately 2% of the population we felt the urge to develop a Dutch PROM for this disease [7]. The version of the COMQ-12 by Van Dinther et al. has already been published as a ‘Dutch’ version [4]. However, there are substantial differences between Flemish and Dutch language. Therefore, in our opinion, the Flemish version is not suitable for the Dutch population. Currently, Bruinewoud et al. are developing a Dutch generic patient related outcome measure consisting of 34 items addressing the severity of ear complaints and their impact on quality of life [1]. According to our opinion, a generic questionnaire is not as useful when studying a well-defined subpopulation, for example a population of patients suffering from CSOM. The to be developed generic ear questionnaire is quite long and therefore time consuming to administer. Both arguments favoured the need for a short disease-specific functional health questionnaire for patients suffering from CSOM with which we are able to detect pre- and post-operative differences in health. With these demands in mind, the Chronic Ear Survey (CES) was potentially very appropriate. The CES is a validated outcomes measure for evaluating the impact of CSOM on adults [8]. This questionnaire was originally validated for the English language and later for the Chinese and Italian language [8, 9]. Our objective was to validate and evaluate the reliability of the Dutch version of the Chronic Ear Survey (CES) in patients suffering from CSOM and to evaluate clinical outcomes of surgery using this questionnaire.

## Methods

### Questionnaire

The CES is a 13 item disease-specific questionnaire, aiming to measure health impact and treatment effectiveness in patients with CSOM (Appendix 1). The questionnaire consists of three domains: activity restriction (AR; 3 items), symptoms (ST; 7 items) and medical resource (MR; 3 items). The answers are ordered as a Likert-type scale varying from 4 to 6 answers per item. The most positive answer is always positioned on the right. A total score is the sum of all 13 items and domain scores are calculated as the sum of concerning domain items. Total score of the CES ranges from 13 to 71, with higher scores indicating better functional health.

### Translation

Permission to translate and validate the original CES was obtained from the Clinical Outcome Research Unit of the Massachusetts Eye and Ear Infirmary [10]. The original CES was translated into the Dutch language independently by the first and the last author. This translation was send to an official translator and native English speaker who retranslated the Dutch version back to the English language. The Dutch and English version were compared by the first, third and last author. A few corrections were made to obtain fluent Dutch sentences without changing the original meaning of the questions. Appendix 2 depicts the Dutch CES (D-CES) after translation.

### Patients and control group

A prospective monocenter observational cohort study was performed at the Amsterdam University Medical Centers (Amsterdam UMC) location AMC between May 2015 and October 2017. Adult patients with CSOM scheduled for ear surgery were asked to complete the D-CES pre- and postoperatively. CSOM was defined as a condition of the middle ear of at least 3 months’ duration and characterised by irreversible pathological changes in the mucosa of the middle ear and/or mastoid and associated with constant or intermittent discharge of bacterial origin [7]. Patient characteristics in terms of age, sex, duration of complaints, number of previous operations, presence of a dry ear postoperatively and results upon pure tone audiometry pre- and postoperatively were collected. Improved postoperative hearing was defined as ≥10 dB improvement of high Fletcher Index (average of 1, 2 and 4 kHz). A decrease of ≥10 dB was used as definition of deterioration of postoperative hearing. Control subjects were also asked to participate in this study. The control group consisted of patients with an eye condition and indication for eye surgery. The study was approved by the medical ethics committee of the Amsterdam UMC and written informed consent was obtained from all patients. Patients in the CSOM group were asked to complete the questionnaire two times preoperatively with at least a two-week-interval in order to investigate test-retest reliability. Approximately 6 months postoperatively all patients completed the questionnaire again. The control group was asked to complete the questionnaire one time preoperatively and once 6 months postoperatively. The D-CES was self-administered, either in the hospital or at home, and if necessary returned by post.

### Internal consistency and test-retest reliability

With regard to the reliability of the D-CES we focused on internal consistency and test-retest reliability of D-CES scores. Internal consistency or homogeneity reflects the statistical coherence of scale items and can be measured using Cronbach’s α coefficient. Cronbach’s α coefficient is based on the weighted average correlation of items within the scale. We also calculated the corrected item-total correlations, which represent the correlation of the single item and the respective total subscale score, excluding that item. Correlations ≥0.30 are considered to be sufficient [11]. Homogeneity and test-retest reliability were investigated preoperatively.

### Known group validity and convergent validity

We investigated two aspects of validity: known-group validity (or clinical validity) and convergent validity. A scale demonstrates known-group validity if it discriminates between (sub) groups of patients with known differences in clinical status. Convergent validity is demonstrated by significant correlations between scale scores and another instrument(s) measuring the same or closely associated heath domains. The known-group validity was assessed by comparing (a) the D-CES baseline scores with baseline scores of our control group and (b) relating the D-CES change scores between baseline and 6 months post-operatively with the duration of preoperative otorrhea, primary or revision surgery, amount of previous operations, presence of a postoperative dry ear, post-operative improved hearing, and postoperative deterioration in hearing. Concerning convergent validity, we compared the pre- and postoperative D-CES scores with correlated subscales of the Dutch version of the Medical Outcome Study 36-Item Short-Form Health Survey (SF-36) which was also administered to all participants [12]. This Quality of Life (QoL) questionnaire was administered twice, once preoperatively and once 6 months postoperatively. The SF-36 consist of eight subscales representing physical (role) functioning, social functioning, emotional (role) functioning, vitality, body pain, and general health perceptions. The scale scores are transformed to a scale of 0 to 100, with a higher score indicating a better QoL. The physical and mental components of the eight subscales can also be combined into a physical and mental component summary score [13]. For this study the social functioning subscale of the SF-36 was compared to the activity subscale of the D-CES, bodily pain to symptoms and general health perceptions to the total questionnaire. No sufficient subscale was present in the SF-36 for comparison with the medical resources subscale of the D-CES.

### Statistics

Patient characteristics were analysed using simple descriptive statistics. Internal consistency was expressed using Cronbach’s α correlation coefficient [14]. Item-total correlations and test-retest reliability were analysed using a Pearson’s correlation coefficient and Intraclass Correlation Coefficient (ICC). ICC values < 0.40 can be considered as poor; between 0.40 and 0.59 as fair; between 0.60 and 0.74 as good; and between 0.75 and 1.00 as excellent [15]. Statistical uncertainty of ICC values was expressed using a 95% confidence interval (CI). Differences between mean (change) scores were analysed using a two-group t test. Convergent correlation patterns were expressed in Pearsons’ rank correlation coefficients. All analyses were performed in Statistical Package for the Social Sciences (SPSS), version 24.

## Results

### Patients and control group

Between March 2015 and October 2016 forty-seven patients with CSOM were included. Twenty-nine out of these 47 patients (62%) fully completed all questionnaires: both the two preoperative and the postoperative questionnaire. In the same period 35 patients in the control group were included. Twenty-six out of 35 control patients (74%) responded to the questionnaire pre- and postoperatively. Table 1 summarizes the patient characteristics. The median age of patients in the CSOM group was 54 compared to 64 in the control group. Median duration of otorrhea was 33 months (range 3 to 380 months).

**Table 1:** Patient characteristics

	Amount of patients (n)	Age, median (range)	Female-to-male ratio	Duration of otorrhea in months, median (range)	Revision surgery n (%)	Previous ear operations, median (range)
CSOM group	29	54 (27–80)	13:16	33 (3–380)	17 (59%)	1 (0–8)
Control group	26	64 (24–92)	15:11	NA	NA	NA

### Internal consistency and test-retest reliability

Internal consistency calculated with Cronbach’s α correlation coefficient of the total scale was 0.69. Homogeneity analyses of the separate domains resulted in α = 0.47 for the AR subscale, α = 0.72 for the ST subscale and α = 0.68 for the MR subscale (Table 2). In general, the items contributed to the reliability of their respective domains. However three relative weak items could be identified; item a3 in the activity restriction domain showed poor correlation with the domain score (item-total correlation is r = 0.10). Removing this item improved the homogeneity of the domain to α = 0.74. Two other weak items were found in the symptom domain (s3, r = 0.11 and s6, r = 0.12). Deletion of these items improved the homogeneity of this subscale to α = 0.76 and α = 0.74, respectively. The Intraclass correlation coefficient (ICC) reflecting test-retest reliability for the total questionnaire was 0.71 (95% CI: 0.44 to 0.86). The ICC for subscale AR was 0.82 (95% CI: 0.65 to 0.92), for subscale ST the ICC was 0.69 (95% CI: 0.42 to 0.85) and for subscale MR it was 0.64 (95% CI: 0.36 to 0.82).

**Table 2:** Homogeneity of the three domains of the D-CES

Domain	Items	α if item deleted	Corrected item-total correlation
Activity restriction	a1. Swimming	.05	.56
α = .47	a2. Keep water out	.30	.33
	a3. Interfering with social activities	.74	.10
Symptoms	s1. Hearing loss	.69	.44
α = .72	s2. Drainage	.65	.59
	s3. Pain	.76	.11
	s4. Odor	.61	.71
	s5. Impact of hearing loss	.66	.51
	s6. Frequency of drainage	.74	.12
	s7. Impact of odor	.63	.62
Medical resource	m1. Doctor visits	.68	.41
α = .68	m2. Oral antibiotics	.59	.48
	m3. Ear drops	.42	.60

### Known group validity and convergent validity

Significant differences were found between the subscale scores and total scores pre- and postoperatively of the CSOM group and the control group, with substantial lower (more impaired) D-CES scores in our target population (Table 3). Also patients with a relative shorter duration of preoperative otorrhea (median < 33 months) had higher mean change of D-CES scores (improved more) compared to patients with longer duration of disease (Table 4). Difference between mean change scores were significant on the subscale AR (*p* = 0.02) and on the total questionnaire (*p* = 0.03). Twenty-four ears (83%) remained dry after surgery objectively. Compared to the patients with persistent discharging ears these patients improved significantly more on the subscales ST and MT, and on the complete questionnaire (*p* values < 0.01). The otorrhea-specific items S2, S6 and M3 all showed significant improvement (*p* <  0.05) postoperatively in patients with dry ears. Twenty-one patients (72%) showed improved hearing postoperatively which resulted in significantly higher change score on each subscale. No significant impact on the D-CES change scores was seen for type of surgery, amount of previous operation and postoperative decreased hearing. Convergent validity of the D-CES demonstrated a moderate correlation between the AR subscale of the D-CES and the social function subscale of the SF-36 in the preoperative situation (*r* = 0.59, Table 5). This correlation was weaker in the postoperative phase (r = 0.29). Weaker correlations were found for ST and the pain subscale of the SF-36 (r = 0.29), and for the total questionnaire and the general health perception subscale of the SF-36 (r = 0.17 and r = 0.37).

**Table 3:** Known-group-validity: D-CES scores in the CSOM group and control group

	tA Mean (SD)	tS Mean (SD)	tM Mean (SD)	tTotal Mean (SD)	pA Mean (SD)	pS Mean (SD)	pM Mean (SD)	pTotal Mean (SD)
CSOM (*n* = 29)	9.59 (3.44)	19.69 (5.86)	9.50 (3.10)	38.79 (8.62)	11.96 (3.43)	29.64 (7.06)	12.45 (2.81)	53.64 (11.90)
Controls (*n* = 26)	15.89 (0.32)	39.40 (1.57)	14.91 (0.43)	70.42 (1.47)	15.39 (2.52)	38.13 (3.95)	14.83 (0.65)	68.35 (5.47)
p=	< 0.001	< 0.001	< 0.001	< 0.001	0.001	0.01	< 0.001	< 0.001

*t* test preoperatively, *p* postoperatively, *A* activity restriction, *S* patient symptoms, *M* use of medical resource

**Table 4:** Known-group-validity: clinical characteristics in relation to mean change scores from baseline to follow-up

		Subscale Activity Restriction	Subscale Symptoms	Subscale Medical Resources	Total questionnaire
Duration of preoperative otorrhea	< 33 months, Mean (SD) *n* = 14	3.92 (3.73)	12.15 (6.53)	4.29 (2.55)	20.23 (8.73)
	≥ 33 months Mean (SD) *n* = 15	0.60 (3.44)	7.33 (8.38)	1.79 (4.42)	10.21 (13.64)
	p	0.02	0.11	0.08	0.03
Primary versus revision surgery	Primary *n* = 12	2.64 (3.41)	9.55 (7.45)	3.50 (3.61)	15.45 (10.33)
	Revision *n* = 17	1.82 (4.25)	9.56 (8.30)	2.69 (3.96)	14.75 (14.00)
	p	0.60	0.99	0.58	0.88
Amount of previous operations	0 operations n = 12	2.64 (3.41)	9.55 (7.45)	3.50 (3.61)	15.45 (10.33)
	≥ 1 operations n = 17	1.82 (4.25)	9.56 (8.30)	2.69 (3.96)	14.75 (14.00)
	p	0.60	0.99	0.58	0.88
Postoperative dry ears	Yes *n* = 24	2.65 (3.71)	11.65 (6.67)	3.79 (3.43)	18.00 (10.16)
	No *n* = 5	−0.20 (4.27)	0.00 (5.48)	−1.50 (2.38)	−2.00 (11.17)
	p	0.14	< 0.01	< 0.01	< 0.01
Postoperative improved hearing	Yes *n* = 21	1.86 (4.56)	15.43 (5.53)	5.00 (1.69)	22.14 (5.11)
	No *n* = 8	2.24 (3.77)	7.62 (7.59)	2.25 (4.10)	12.55 (13.33)
	p	0.83	0.02	0.02	0.01
Postoperative decreased hearing	Yes n = 5	2.00 (2.83)	7.00 (5.57)	1.20 (3.56)	8.20 (13.31)
	No n = 24	2.17 (4.14)	10.13 (8.24)	3.43 (3.76)	16.59 (11.98)
	p	0.93	0.43	0.24	0.17

**Table 5:** Convergent validity of the D-CES and its domains

D-CES domain	Matching health domain SF-36	Correlation coefficient preoperatively	Correlation coefficient postoperatively
Activity restriction	Social functioning subscale	0.59	0.29
Symptoms	Bodily pain subscale	0.29	0.29
Medical resources	NA	–	–
Total questionnaire	General Health perceptions subscale	0.17	0.37

*NA* not applicable since there is no correlating SF-36 subscale

## Discussion

The D-CES is a suitable questionnaire to obtain subjective data of patients with CSOM. The Dutch otologist is now able to use this questionnaire as a disease specific tool to evaluate pre- and postoperative satisfaction. As the CES is now available in four languages it facilitates easier comparison of different study populations [8–10]. Forward-backward translation of the original CES into the Dutch language was performed similar to other studies [2, 4]. A few items of the CES have a 6-point Likert-type response scale. This poses a limitation as it denies the option ‘no change’ within the answers. Questionnaires like the Zurich chronic middle ear inventory (ZCMEI-21) specifically chose a 5-point response option to address this problem [3] compared to the original Chronic Otitis Media Questionnaire-12 (COMQ-12) which comprises of a 6-point Likert scale [16]. At this moment the COMQ-12 is translated into Flemish, Russian and Serbian [4, 17, 18]. Surprisingly it appears that the Russian version is based on a 5-point Likert scale. In that case we believe that these questionnaires are not comparable anymore. Recently Phillips et al. developed the Chronic Otitis Media Benefit Inventory (COMBI) [19]. This is a mixed generic and specific dynamic patient-reported outcome measure. It is derived from the COMQ-12 and asks patients to report any perceived changes in symptoms so it can only been used following an intervention. We believe that it is important to start with baseline subjective data before surgery to enable comparison of disease specific patient reported outcomes. As the original CES was already validated in a large population we chose a relatively small population for the Dutch validation [10]. This is in concordance with the translation and validation as performed by Van Dinther et al. [4]. In our opinion patients with eye disease are very similar to patients with ear disease and the postoperative burden is also comparable. Therefore these patients were suitable to serve as control subjects. Internal consistency of the D-CES was not as good as the original English version [10]. Especially the alpha coefficient of the AR subscale was relative low. As the Cronbach’s α values partly depends on the number of items on a scale, the internal consistency of the questionnaire was additionally described in terms of corrected item-total correlations. The low item-total correlation of item A3 can probably explain that this item reflects social function instead of physical activities. Still the homogeneity coefficient of the total questionnaire is acceptable. The point estimates of the test-retest reliability (of the subscales) of the D-CES turned out to be good. Joseph et al. did not find significant associations between clinical parameters and functional outcomes, using the Glasgow Benefit Inventory (GBI) [5]. Using the disease-specific D-CES we found that improvement scores were significantly influenced by preoperative duration of complaints, postoperative dry ears and postoperative improved hearing. These findings contribute to the clinical validity of the D-CES. Correlation between D-CES and SF-36 subscales were only moderate to small, showing no substantial correlation between a disease-specific functional measure and a generic measure quality of life, as suspected. A limitation of this study is that a number of clinimetric properties was not evaluated and are thus unsettled. This include the assessment of convergent validity with a disease-specific measure, a more in-depth investigation of the responsiveness of the D-CES to measure health changes over time, and – related to this issue - the determination of the smallest D-CES change score (after treatment) that is considered clinically important for the patient.

## Conclusions

The Dutch version of the Chronic Ear Survey provides an appropriate short, easy to administer, disease specific questionnaire to assess patient’s perceived functional health in CSOM. The CES is now validated in four languages with good potential of population comparison. The D-CES showed acceptable homogeneity and good test-retest reliability. Known-group (or clinical) validity demonstrated a clear association with preoperative duration of complaints, postoperative dry ears and postoperative improved hearing. These findings suggest that the D-CES can be used to evaluate surgical outcome as well.

## Data Availability

The datasets used and/or analysed during the current study are available from the corresponding author on reasonable request.

## Appendix 1

**Table 6:** The original Chronic Ear Survey (CES) [8]

Activity Restriction Based-Subscale						
A1	Because of your ear problem, you don’t swim or shower without protecting your ear.					
	*Definitely true*	*True*	*False*	*Definitely false*		
A2	At the present time, how severe a limitation is the necessity to keep water out of your ears?					
	*Very severe*	*Severe*	*Moderate*	*Mild*	*Very mild*	*None*
A3	In the past four weeks, has your ear problem interfered with your social activities with friends, family and groups?					
	*All of the time*	*Most of the time*	*A good bit of the time*	*Some of the time*	*A little of the time*	*None of the time*
Symptom scale						
S1	Your hearing loss is:					
	*Very severe*	*Severe*	*Moderate*	*Mild*	*Very mild*	*None*
S2	Drainage from your ear is:					
	*Very severe*	*Severe*	*Moderate*	*Mild*	*Very mild*	*None*
S3	Pain from your ear is:					
	*Very severe*	*Severe*	*Moderate*	*Mild*	*Very mild*	*None*
S4	Odor from your ear is very bothersome to you and/or others:					
	*Definitely true*	*True*	*Don’t know*	*False*	*Definitely false*	
S5	The hearing loss in your affected ear bothers you:					
	*All of the time*	*Most of the time*	*A good bit of the time*	*Some of the time*	*A little of the time*	*None of the time*
S6	In the past 6 months, please estimate the frequency that your affected ear has drained:					
	*Constantly*	*5 or more times, but not constantly*	*3–4 times*	*1–2 times*	*Not at all*	
S7	The odor from your ear affected ear bothers you and/or others:					
	*All of the time*	*Most of the time*	*A good bit of the time*	*Some of the time*	*A little of the time*	*None of the time*
Medical Resource Subscale						
M1	In the past 6 months, how many separate times have you visited your doctor, specifically about your ear problem?					
	*More than 6 times*	*5–6 times*	*3–4 times*	*1–2 times*	*None*	
M2	In the past 6 months, how many separate times have you used oral antibiotics to treat your ear infection?					
	*More than 6 times*	*5–6 times*	*3–4 times*	*1–2 times*	*None*	
M3	In the past 6 months, how many separate times have ear drops been necessary to treat your ear condition?					
	*More than 6 times*	*5–6 times*	*3–4 times*	*1–2 times*	*None*	

Copyright© 1997 Massachusetts Eye and Ear Infirmary and Outcome Science, Lic

## Appendix 2

**Table 7:** The Dutch Chronic Ear Survey (D-CES)

Schaal gebaseerd op activiteitenbeperking						
A1	Vanwege uw oorprobleem zwemt of doucht u niet zonder uw oor te beschermen.					
	*Helemaal juist*	*Grotendeels juist*	*Grotendeels onjuist*	*Helemaal onjuist*		
A2	Hoe erg belemmerend is op dit moment de noodzaak om water uit uw oren te houden?					
	*Heel erg*	*Erg*	*Gemiddeld*	*Matig*	*Zeer matig*	*Niet*
A3	Heeft uw oorprobleem u in de afgelopen 4 weken gehinderd in uw sociale activiteiten met vrienden, familie of groepen?					
	*Altijd*	*Bijna altijd*	*Meestal*	*Regelmatig*	*Soms*	*Nooit*
Schaal gebaseerd op symptomen						
S1	Uw gehoorverlies is:					
	*Zeer groot*	*Groot*	*Gemiddeld*	*Matig*	*Mild*	*Geen*
S2	Uw loopoor is:					
	*Zeer ernstig*	*Ernstig*	*Gemiddeld*	*Matig*	*Mild*	*Geen*
S3	Uw oorpijn is:					
	*Zeer ernstig*	*Ernstig*	*Gemiddeld*	*Matig*	*Mild*	*Geen*
S4	De geur uit uw oor is erg vervelend voor u en/of anderen:					
	*Helemaal juist*	*Grotendeels juist*	*Ik weet het niet*	*Grotendeels onjuist*	*Helemaal onjuist*	
S5	Het gehoorverlies in uw aangedane oor stoort u:					
	*Altijd*	*Meestal*	*Een groot deel van de tijd*	*Een deel van de tijd*	*Een klein deel van de tijd*	*Nooit*
S6	Wilt u de frequentie schatten dat u in de afgelopen 6 maanden een loopoor hebt gehad aan de aangedane zijde?					
	*Constant*	*5 of meer keer, maar niet constant*	*3–4 keer*	*1–2 keer*	*Helemaal niet*	
S7	De geur uit uw aangedane oor stoort u en/of anderen:					
	*Altijd*	*Meestal*	*Een groot deel van de tijd*	*Een deel van de tijd*	*Een klein deel van de tijd*	*Nooit*
Schaal gebaseerd op medische zorg						
M1	Hoeveel afzonderlijke keren hebt u in de afgelopen 6 maanden uw arts bezocht specifiek vanwege uw oorprobleem?					
	*Meer dan 6 keer*	*5–6 keer*	*3–4 keer*	*1–2 keer*	*Niet*	
M2	Hoeveel afzonderlijke keren hebt u in de afgelopen 6 maanden orale antibiotica gebruikt om uw oorinfectie te behandelen?					
	*Meer dan 6 keer*	*5–6 keer*	*3–4 keer*	*1–2 keer*	*Niet*	
M3	Hoeveel afzonderlijke keren waren in de afgelopen 6 maanden oordruppels nodig om uw ooraandoening te behandelen?					
	*Meer dan 6 keer*	*5–6 keer*	*3–4 keer*	*1–2 keer*	*Niet*	

Copyright© 1997 Massachusetts Eye and Ear Infirmary and Outcome Science, Lic

## Abbreviations

Amsterdam UMC: Amsterdam University Medical Centers
AR: Activity Restriction
CES: Chronic Ear Survey
CI: Confidence Interval
COMBI: Chronic Otitis Media Benefit Inventory
COMQ-12: Chronic Otitis Media Questionnaire-12
CSOM: Chronic Suppurative Otitis Media
CT: Computer Tomography
D-CES: Dutch version of the Chronic Ear Survey
GBI: Glasgow Benefit Inventory
ICC: Intraclass Correlation Coefficient
MR: Medical Resources
NA: Not applicable
PROM: Patient Reported Outcome Measure
QoL: Quality of Life
SD: Standard Deviation
SF-36: Short Form 36
SPSS: Statistical Package for the Social Sciences
ST: Symptoms
ZCMEI-21: Zurich chronic middle ear inventory
